# Gap Arthroplasty versus Interpositional Arthroplasty for Temporomandibular Joint Ankylosis: A Meta-Analysis

**DOI:** 10.1371/journal.pone.0127652

**Published:** 2015-05-26

**Authors:** Junli Ma, Limin Liang, Hua Jiang, Bin Gu

**Affiliations:** 1 Department of Stomatology, Guangzhou General Hospital of Guangzhou Military Command, Guangzhou, Guangdong, China; 2 Department of Stomatology, Chinese PLA General Hospital, Beijing, China; University of North Carolina at Chapel Hill, UNITED STATES

## Abstract

Gap arthroplasty (GA) and interpositional arthroplasty (IA) are widely used for the treatment of temporomandibular joint ankylosis (TMJA). However, controversy remains as to whether IA is superior to GA. PubMed, EMBASE, the Cochrane Library, the Web of science and the China National Knowledge Infrastructure were searched for literature regarding these procedures (published from 1946 to July 28, 2014). A study was included in this analysis if it was: (1) a randomized controlled trial or non-randomized observational cohort study; (2) comparing the clinical outcomes between GA and IA with respect to the maximal incisal opening (MIO) and reankylosis; (3) with a follow-up period of at least 12 months. The methodological quality of the included studies was evaluated according to the Newcastle-Ottawa Scale Eight non-randomized observational cohort studies with 272 patients were included. All the statistical analyses were performed using the RevMan 5.3 and Stat 12. The pooled analysis showed no significant difference in the incidence of reankylosis between the IA group (13/120) and the GA group (29/163) (RR= 0.67, 95% CI=0.38 to 1.16; Z=1.43, p=0.15). The IA group showed a significantly larger MIO than the GA group (MD=1.96, 95% CI=0.21 to 3.72, Z=2.19, p=0.03, I^2^=0%). In conclusion, patients with TMJA could benefit more from IA than GA, with a larger MIO and a similar incidence of reankylosis. IA shows to be an adequate option in the treatment of TMJA based on the results of maximal incisal opening.

## Introduction

Temporomandibular joint ankylosis (TMJA) is characterized by a limited range of motion of the mandible, which leads to difficulties in mastication, impairment of speech, facial deformity and the resulting psycho-social problems, especially in children. TMJA is caused mostly by trauma and infection, TMJ surgery and systemic diseases like rheumatoid arthritis are also possible causes [1–3].

Surgery is the treatment of choice for TMJA, aiming to restore joint function and prevent reankylosis. Three main surgical techniques are currently employed: (1) Gap arthroplasty (GA); (2) Interpositional arthroplasty (IA); and (3) Reconstruction of the articulation (RA) after resection of the ankylotic mass with autogenous or alloplastic grafts [4–7].

GA is the oldest surgical technique used in treating TMJA, and is technically less demanding with shorter operation time and lower cost than IA, but it has been associated with a higher incidence of reankylosis [8]. IA is believed to be able to reduce the occurrence of reanykylosis by insertion of interpositional materials after resection of the ankylosis. However, the results of studies comparing GA and IA are controversial [9–14], and the debate concerning which technique is better remains [9, 15–17]. This status makes the selection of the most appropriate surgery difficult for surgeons.

We performed this meta-analysis to compare the clinical outcomes of GA and IA in order to answer the question that which technique should be employed, GA or IA, for patients with TMJA. Our hypothesis was that IA would provide a lower incidence of reankylosis and larger mouth opening than GA. The meta-analysis of observational studies in epidemiology (MOOSE) guideline was used to conduct this analysis [18].

## Materials and Methods

### Search strategy

PubMed, EMBASE, Cochrane Library, the Web of science and the China National Knowledge Infrastructure were searched using combinations of the following search terms: temporomandibular joint, TMJ, ankylosis, ankylosis resection, condylectomy, osteoarthrectomy, arthroplasty, gap arthroplasty, interpositional arthroplasty, temporal muscle and fascia flap, temporalis muscle, temporalis myofascial flap, temporalis fascia, and temporalis superficial fascia flap. The search included studies published from 1946 to July 28, 2014. No restrictions for the publication date or language were applied. All the relevant reports were screened using the title and abstract, the full text of potentially relevant studies was retrieved to evaluate the eligibility of the study. A manual search was performed by screening the reference lists of eligible studies to prevent any omissions. The “British Journal of Oral and Maxillofacial Surgery,” “International Journal of Oral and Maxillofacial Surgery,” “Journal of the American Dental Association,” “Journal of Cranio-Maxillofacial Surgery,” “Journal of Dental Research,”“Journal of Oral and Maxillofacial Surgery,” “Oral Diseases,” and “Oral Surgery, Oral Medicine, Oral Pathology, Oral Radiology, and Endodontology” were also manually searched for relevant reports.

### Inclusion criteria

A study was selected if it was: (1) a randomized controlled trial (RCT) or observational cohort study; (2) comparing the clinical outcomes between GA and IA. GA was defined as the resection of ankylosis without the use of interposition material to fill in the space between the articular cavity and the mandibular ramus, and IA was defined as resection of ankylosis with the insertion of biological or non biological materials into the space; 3) with a follow-up period of at least 12 months and 4) outcome data including the maximal incisal opening (MIO) and reankylosis.

The potentially eligible studies were assessed independently by three authors (Ma Junli, Gu Bin, Jiang Hua). Any uncertainty regarding eligibility was discussed and the decision regarding whether to include it was based on a consultation with the fourth reviewer (Liang Limin).

### Data extraction

An Excel (Microsoft, Redmond, USA) sheet was designed to record the following information: the year of publication, name of the first author, study design, age of the patients at the time of the operation, interventions performed, sample size, the interpositional materials used in IA, follow-up duration, postoperative MIO, and reankylosis rate.

### Quality assessment

The methodological quality of the included studies was independently evaluated by 2 observers using the Newcastle-Ottawa Scale (NOS), which was specifically designed to evaluate the quality of non-randomized studies like cohort studies, and the assessment outcomes are listed in Table 1.

**Table 1:** The characteristics of the studies included in the meta-analysis.

Author(Year)	Sample size(IA/GA)	IA material	Mean age at operation(Yrs)	Follow-up (Ms)	Incidence of reankylosis (IA/GA)	Mean MIO (IA/GA, mm)	Study quality (Max = 9)
Tanrikulu R(2005)	9/8	TMF	12	12–180	11.1% (1/9)/0% (0/8)	32.1/31.0	7
Hu TX(2005)	14/41	TSF+other	17	4–180	28.6%(4/14)/24.4% (10/41)	NA/NA	6
Erol B (2006)	15/34	TMF	unknown	12–144	0% (0/15)/8.8% (3/34)	NA/NA	7
Danda Ak (2009)	8/8	TMF	9.6	26.5	12.5% (1/8)/12.5% (1/8)	31.4/31.1	7
Zhi K(2009)	12/25	TSF+disc	22.3	12–132	0% (0/12)/12% (3/25)	NA/NA	6
Elgazzar RF(2010)	14/11	TMF	unknown	14–96	7.1% (1/14)/18.2% (2/11)	30.7/29.1	6
Ramezanian (2006)	26/22	unknown	19.5	59	23.1%(6/26)/45.4% (10/22)	33.9/32.1	6
Holmlund (2013)	22/14	TMF	49	12–108	0% (0/22)/0% (0/14)	36.7/30.9	6

Ms: months; Yrs: years; mm: millimeter

### Assessment of heterogeneity

The heterogeneity was tested using Chi-squared and I-squared tests. The heterogeneity was considered to be significant if *P*<0.05 for the chi-squared test, and the I-squared statistic was defined as follows: 0 to 24% = no heterogeneity, 25 to 49% = moderate heterogeneity, 50 to 74% = large heterogeneity; and 75 to 100% = extreme heterogeneity.

### Outcome measures

The two outcomes of interest were the maximum incisal opening (MIO, measured in millimeters) and the incidence of reankylosis.

### Statistical analysis

The risk ratio (RR) with a 95% confidence interval (95% CI) was calculated for binary outcomes, and the mean difference (MD) with 95% CI was calculated for continuous outcomes. A fixed effects model (Mantel-Haenszel method) was used if there was no heterogeneity (*p* >0.05 or I-squared ≤24%), otherwise a random effects model (Der Simonian-Laird method) was used. P<0.05 was considered to be statistically significant. The publication bias was evaluated using a funnel plot and Egger’s test. Moreover, a sensitivity analysis was applied for the incidence of reankylosis based on the leave-one-out approach. All statistical analyses were performed using the RevMan 5.3 (Cochrane Collaboration, Software Update, Oxford, UK) and Stat 12 (StataCorp, College Station, TX).

## Results

### Literature search

**Fig 1:**
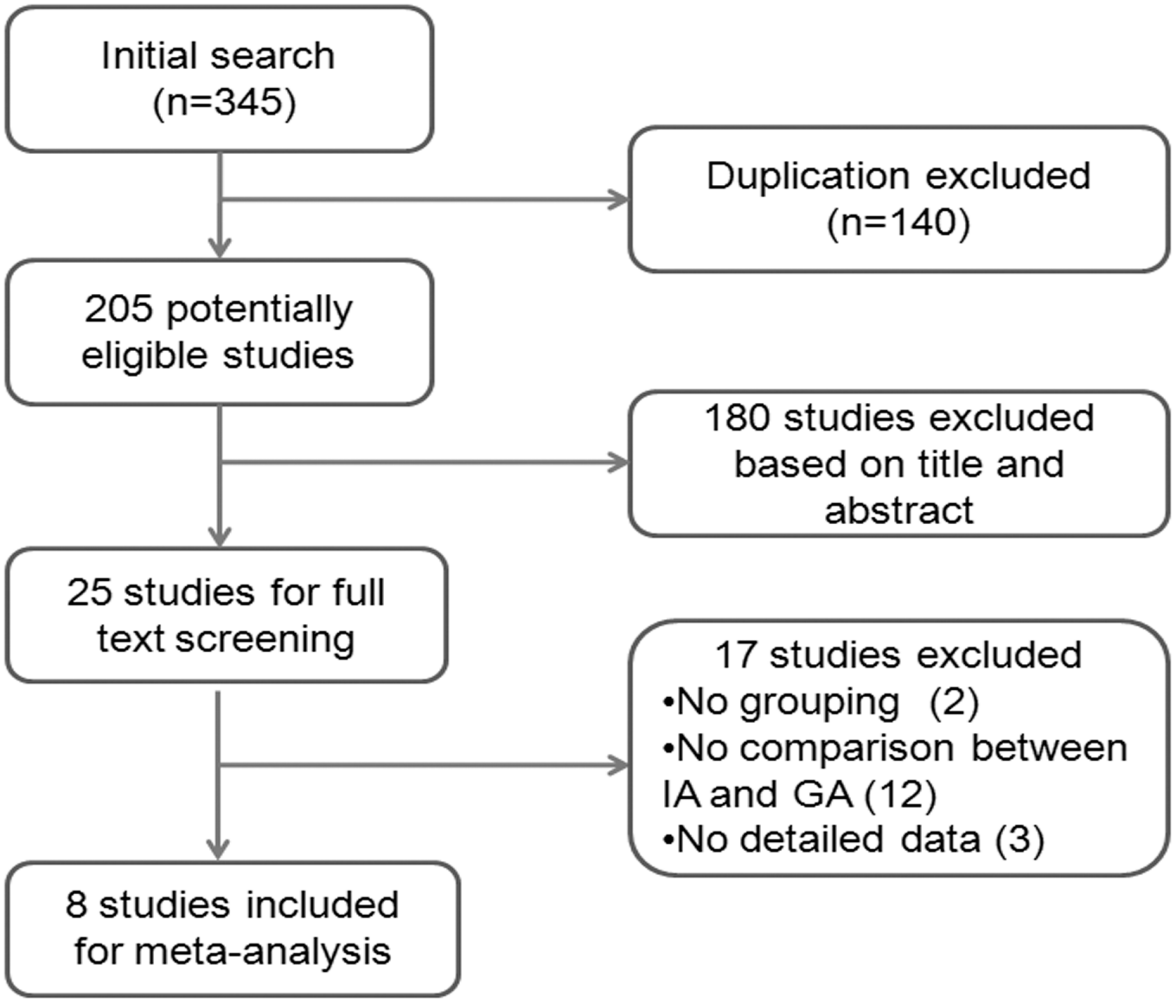
Flow diagram of the study selection.

The initial search identified 345 records, and after the exclusion of duplicate publications and evaluation of the titles and abstracts, 320 studies were excluded. The full text of 25 potentially eligible publications was further screened according to the inclusion criteria. Finally, eight studies with a total of 272 patients were included in the meta-analysis (Fig 1) [9–11, 14, 19–22]. All of these studies were retrospective studies, and no RCTs were found. The mean age of patients at the time of operation was 21.6 years old. A temporalis muscle flap (TMF) was chosen as the interpositional material in most studies (5/8), and a temporalis superficial fascia flap (TSF) was used in 2 studies. Three studies scored 7 according to the NOS, while the other 5 studies scored 6 (Table 1).

### The primary outcome: reankylosis

**Fig 2:**
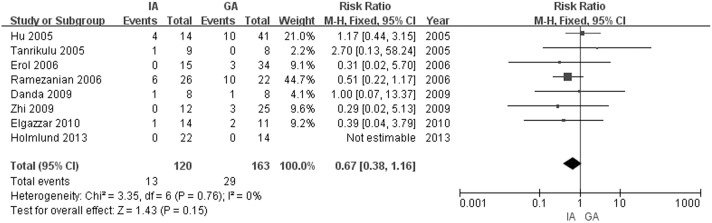
Forest plot of the occurrences of reankylosis (GA vs. IA).

Eight studies with a total of 272 patients reported the information about incidence of reankylosis [9–11, 14, 19–22]. Fig 2 shows a forest plot of the incidence of reankylosis between the patients who underwent GA and those who underwent IA. Neither the Chi-squared nor the I-squared test showed significant heterogeneity (Q = 3.35, df = 6, p = 0.76; I^2^ = 0%), so a fixed-effects model was used. Four studies reported a higher incidence of reankylosis in the GA group [10, 11, 14, 20], and the IA group showed higher incidence of reankylosis in 2 studies [9, 19], the other 2 studies showed an equal incidence of reankylosis [21, 22]. The IA group had a tendency to have a lower incidence of reanylosis (13/120) than the GA group (29/163), but the difference was not significant (RR = 0.67, 95% CI = 0.38 to 1.16; Z = 1.43, p = 0.15; Fig 2).

**Fig 3:**
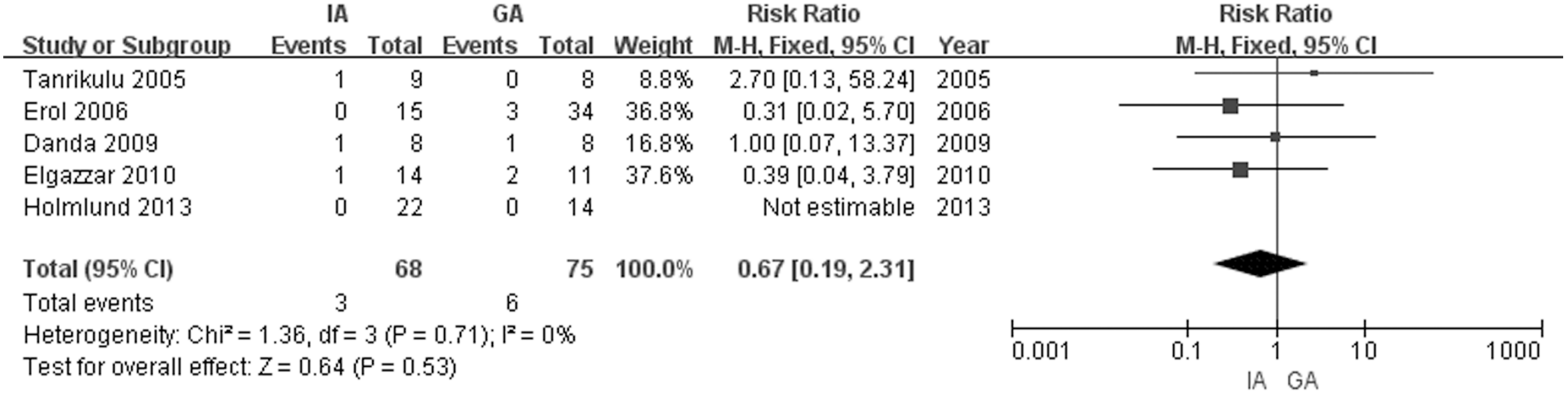
Forest plot of the occurrences of reankylosis—GA vs. IA (TMF).

Among the 8 studies included in the above analysis, a single TMF was used as the interpositional material in 5 studies [9, 10, 20–22]. We extracted the related data from these studies, and performed a further comparison with the GA group to rule out the impacts of different interpositional materials on the incidence of reankylosis. No significant heterogeneity was found among the studies (Q = 2.53, df = 4, P = 0.64; I^2^ = 0%). There were 3/68 cases with reankylosis in the IA (TMF) group and 6/75 in the GA group, and the difference between the IA (TMF) and GA groups in was not significant. (RR = 0.67, 95% CI = 0.19 to 2.31, Z = 0.64, p = 0.53; Fig 3).

### The secondary outcome (the MIO)

**Fig 4:**
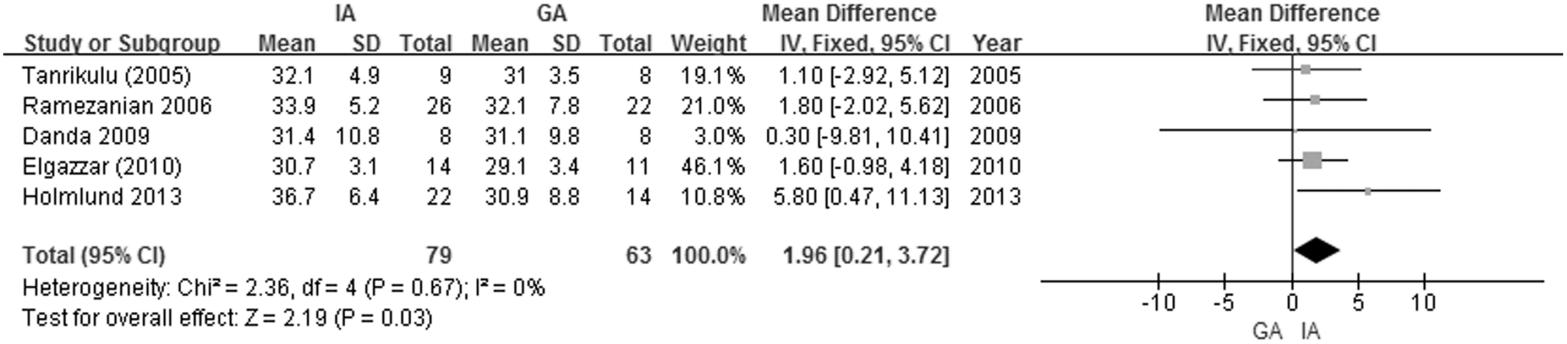
Forest plot of the MIO (GA vs. IA).

Five studies with a total of 142 patients provided the data necessary to compare the MIO between the GA and IA groups [9–11, 21, 22]. No significant heterogeneity was found among these studies (Q = 1.95, df = 4, P = 0.75; I^2^ = 0%), so a fixed-effects model for the meta-analysis of MIO was used. The IA group showed a significantly larger MIO than the GA group (33.0 mm versus 30.8 mm, MD = 1.96, 95% CI = 0.21 to 3.72, Z = 2.19, p = 0.03, I^2^ = 0%, Fig 4). Four of these 5 studies used TMF as interpositional material [9, 10, 21, 22], and the type of material was unclear in the other one study [11]. We excluded this study from a comparison in MIO between IA (TMF) and GA group, and found that the IA (TMF) group also showed a larger MIO than GA group with a p value of 0.05 (MD = 2.01, 95% CI = 0.03 to 3.98, Z = 1.99, p = 0.05, I^2^ = 0%).

### Publication bias

**Fig 5:**
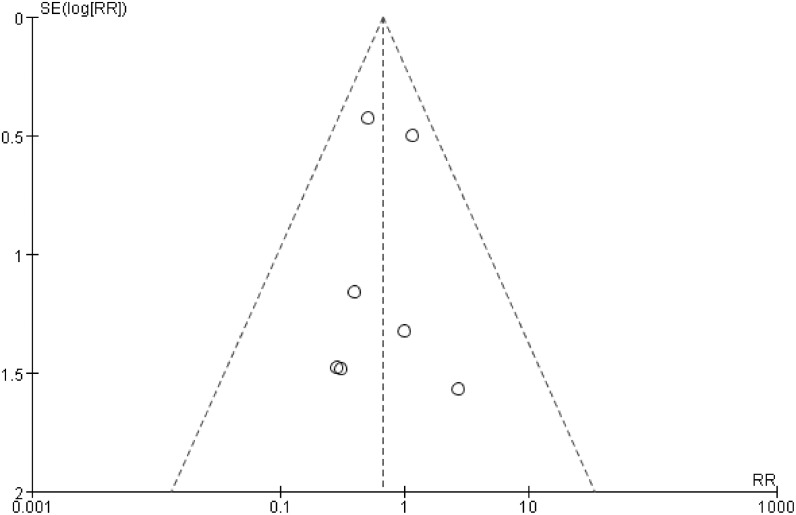
Funnel plot of the occurrences of reankylosis in the GA and IA groups, Egger’s test results: t = -0.12, p = 0.912.

**Fig 6:**
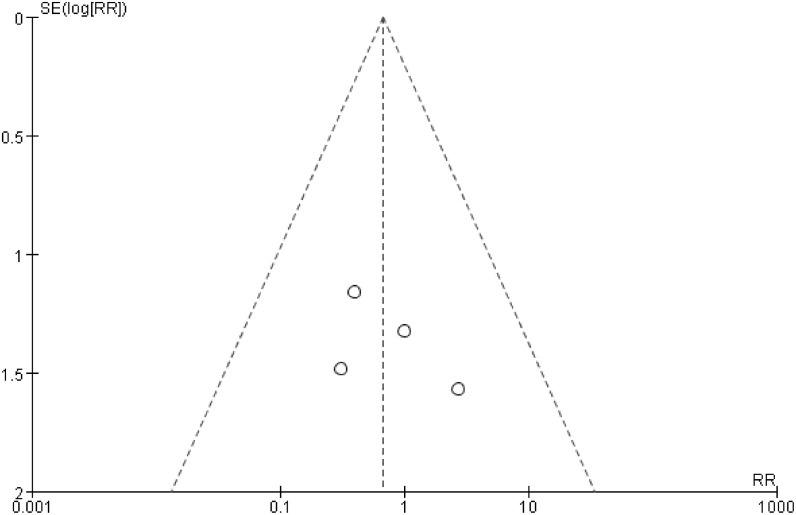
Funnel plot of the occurrences of reankylosis in GA and IA (TMF) groups, Egger’s test results: t = 0.91, p = 0.458.

**Fig 7:**
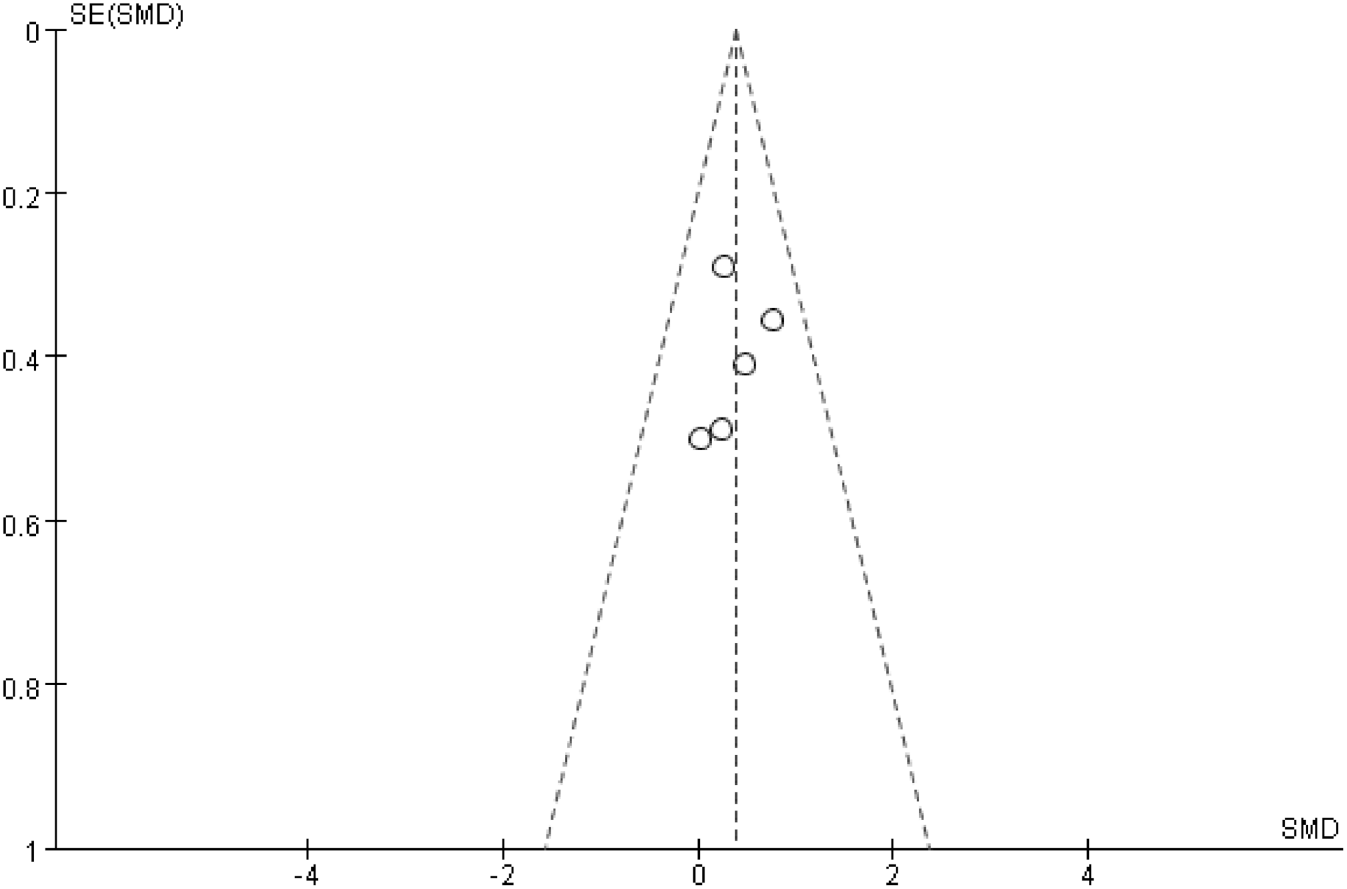
Funnel plot of the MIO of GA and IA groups, Egger’s test results: t = -0.48, p = 0.666.

The symmetric funnel-shaped distribution and the results of Egger’s test showed that there was no significant publication bias in the above analyses (Figs 5, 6 and 7).

### Sensitivity analysis

**Fig 8:**
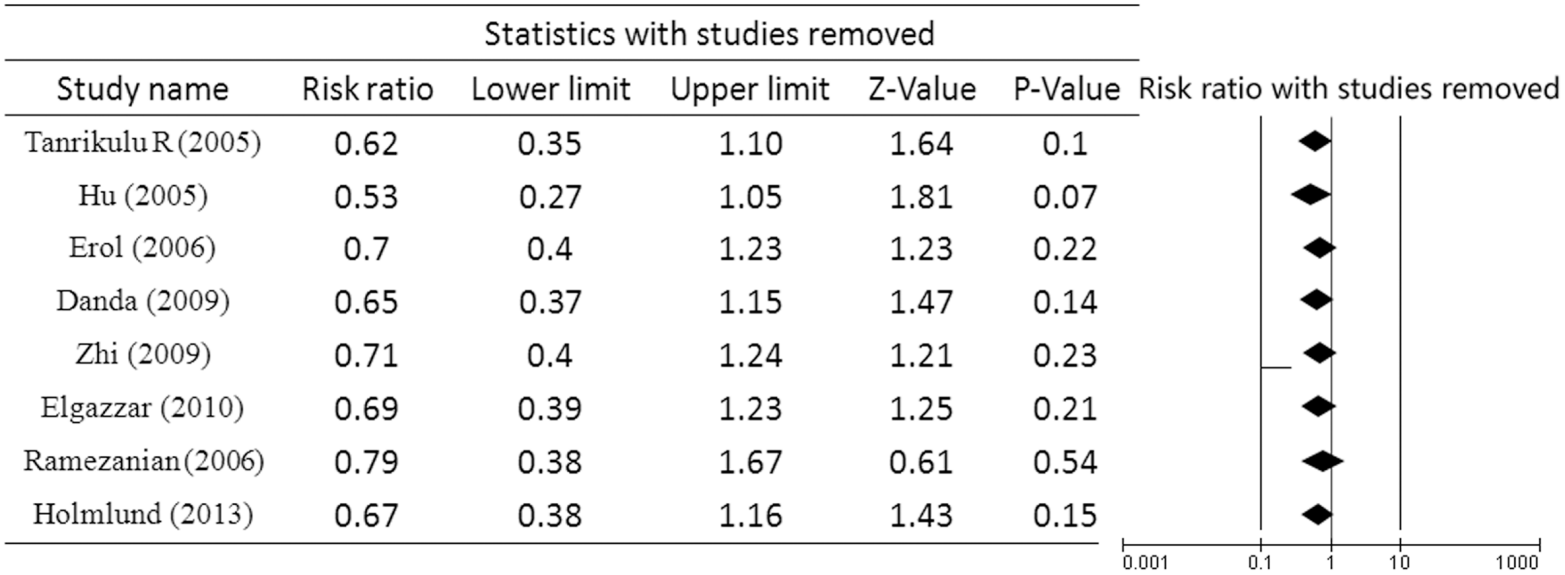
Sensitivity analysis of the influence of individual studies on the pooled estimates for reankylosis.

After one study was removed in turn, the direction and magnitude of the combined estimates of reankylosis did not have a large variation (Fig 8). This result indicated that the present meta-analysis had good reliability.

## Discussion

In this meta-analysis, 8 studies were included for the comparison of reankylosis and the postoperative MIO between those who underwent GA and IA. The pooled results showed that both GA and IA could effectively improve MIO, but neither GA nor IA could completely prevent reankylosis. Our first hypothesis that IA could have a lower incidence of reankylosis than GA was rejected, the pooled data did not reveal significant difference between GA and IA with respect to reankylosis, though IA did show a trend towards lower incidence. The second hypothesis that IA could result into a significantly larger MIO than GA was accepted, the IA group showed a significantly larger MIO than GA.

TMJA has remained a challenge for surgeons since the first condylectomy was performed almost 200 years ago, in 1826 by Humprey [23], mostly because of reankylosis, the most frustrating outcome. IA was first performed later by Verneuil in 1860 [12]. Then, articulation reconstruction and distraction osteogenesis were introduced to restore the function of TMJ and prevent reankylosis [24, 25]. Among these four techniques, GA and IA are the most widely used techniques. Although they have been performed for more than 150 years with many improvements, the argument as to which technique is superior is still ongoing [26].

GA has its own advantages, such as the simple operation, shorter operation time, and low cost [16]. However, GA has been associated with a higher incidence of reankylosis. In an early report published by Topazian in 1964, a recurrence rate as high as 53% was observed in patients who underwent GA, so he recommended that IA should be used as preferred treatment for TMJA [8, 26].

IA is an improvement of GA that involves the insertion of interpositional materials (autogenous and alloplastic) into the space created by GA to prevent reankylosis [4–7]. Although some studies have shown that IA is superior to GA [9, 14], other authors have claimed that GA can also provide satisfactory outcomes [16, 17]. In Roychoudhury’s [2] study, reankylosis was observed in only 2% of the patients who underwent GA. Some authors believe that wide gap arthroplasty followed by vigorous physiotherapy is sufficient to prevent recurrence [27], and the reason for higher incidence of reankylosis of GA is mostly due to the insufficient resection of the ankylosis [28]. In a well-designed retrospective study, Danda et al. [21] did not find significant any differences in the MIO and incidence of reankylosis between the patients who underwent GA and those who received IA, and he concluded that the success of treatment depended on patient cooperation, active physiotherapy, and regular follow-up.

In the present analysis, the pooled data did not show a significantly higher incidence of reankylosis in patients who underwent GA than in those who underwent IA. We also selected the studies in which only TMF was used as the interpositional material to exclude the impact of different materials on the occurrence of reankylosis, and the results were still similar, with no significant differences detected between GA and IA.

Reankylosis or a loss of range of motion mostly occurs within the first year postoperatively, varying from 1 month to 13 years after surgery [12, 19, 22, 29]. The reasons for reankylosis vary, but the major cause is thought to be inadequate resection of the ankylosis and poor compliance of the patient with postoperative mouth-opening exercises or physiotherapy. Other factors that can lead to reankylosis include wound infection, and a foreign body reaction caused by interpositional materials [19, 30, 31].

In contrast with an early meta-analysis performed by Katsnelson et al. [32], we did not treat MIO as our primary measurement, because most studies have shown that the difference in the MIO between GA and IA was usually small, at just a few millimeters. Our results did reveal that IA can achieve a significantly larger MIO than GA, but the difference was only 1.96 mm, so the MIO might not be a decisive factor influencing the surgeon to choose IA over GA.

With regard to the interpositional material, TMF is the most widely used, as shown in the present analysis. This is due to its close proximity to the surgical site, easy usage, rich blood supply and minimal donor-site morbidity [21, 33]. However, using the TMF is also associated with several potential disadvantages, such as scar contracture and resorption. Other materials, like dermis-fat grafts, can also achieve satisfying results in terms of mouth-opening, jaw function, and reankylosis [30, 34, 35]. We also made an attempt to evaluate the efficacy of these materials by a meta analysis, limited eligible studies precluded this attempt.

Postoperative occlusion and mandible movement are also important factors that should be considered before surgery. A gap wider than 10 mm is believed to be mandatory to prevent reankylosis during the practice of GA [10, 12, 14, 22]. However, such a wide gap could lead to shortened mandibular ramus, which might result in mandible deviation and malocclusion, such as open bite and premature occlusion [36, 37]. In contrast, IA require a narrower gap, often <5 mm, and when this narrower gap is combined with interpositional material, the TMJ anatomic structure could be better restored, which would reduce the risk of mandible deviation and malocclusion [30,31, 34].

We tried to summarize clear indications for each technique based on the literatures. However, the results were disappointing. Most studies did not give a clear or specific description of the indication, just as Gaurav Jain et al. encountered in his study, reflecting the uncertainty or variability of the selection of surgery [12]. Therefore, an exclusive indication for GA or IA might be unavailable or unnecessary because there are great overlaps between their coverage, especially when there is no significant loss of mandibular ramus height.

Taking reankylosis, the MIO and the function of mandible into consideration, we recommended IA as the first choice for treating TMJA. It is especially recommended for patients with reankylosis after initial GA. If a loss of the ramus height could be fully restored by interpositional materials, IA should also be selected, otherwise, joint reconstruction with autogenous or alloplastic materials is considered to provide better results.

IA also has its own disadvantages, including the time consuming procedure, sophisticated nature of technique, the donor site morbidity, and the risk of resorption of and foreign body reaction against interpositional materials. All of these factors, plus the surgeon’s experiences, the patients’ age, the patient’s ability to tolerate surgery and their likely cooperation with postoperative exercises should be considered when selecting a specific surgical modality [38, 39].

Regardless of which technique is selected, the following are recommended to prevent reankylosis: 1) wide surgical exposure and complete resection of ankylosis; 2) the use of appropriate interposition materials, with TMF recommended by most authors; 3) early mobilization, usually begin within the first week after surgery; 4) intermaxillary fixation (wire or elastic) is not recommended; 5) regular physiotherapy; and 6) good cooperation from the patient and close follow-up [4, 13, 14, 16, 20, 40].

The first limitation of our study is the inability to use RCTs to perform the meta-analysis. Indeed, because of the low incidence of TMJA and various factors affecting the outcomes, together with clinical difficulties, it is hard to perform a RCT with a large sample size. Therefore, although suboptimal, observational studies may be the most feasible study design. Meta analyses using observational studies according to the MOOSE guideline can provide great enhancement of clinical studies and are becoming popular [41, 42]. Another limitation of our study is the quality of the included studies, most of which rated 6 or 7 according to the NOS. Most studies lacked strict grouping criteria and detailed information on the measurement. With these shortcomings, the reader should be cautious when interpreting our results. To obtain more convincing results, the following improvements would be needed: 1) selecting patients according to a common TMJA classification and with a clear description of the indications for different surgeries; 2) performing a multicenter study to increase the sample of patients; 3) detailed documentation of the factors related to the evaluation, including the classification, gap distance, postoperative occlusion, mandible movement, and physiotherapy; and 4) applying blind measurement and grouping if possible.

## Supporting Information

S1 Checklist PRISMA 2009 Checklist. (DOC)

S1 Text The PubMed Search strategy. (DOC)
