# Supplementary material for: Gap Arthroplasty versus Interpositional Arthroplasty for Temporomandibular Joint Ankylosis: A Meta-Analysis
Source: PLoS One. 2015 May 26;10(5):e0127652. doi: 10.1371/journal.pone.0127652 (PMC4444315; doi:10.1371/journal.pone.0127652)
Supplement: S1 Text — (DOC) [file pone.0127652.s002.doc]

**PubMed search strategy**

**Key words:** temporomandibular joint; TMJ; ankylosis; gap arthroplasty; interpositional arthroplasty; interposition arthroplasty; ankylosis resection; condylectomy; osteoarthrectomy; temporal muscle and fascia flap; temporalis muscle; temporalis myofascial flap; temporalis fascia; Temporalis superficial fascia flap

**Search strategy:** (((((((ankylosis) AND ((temporomandibular joint) OR TMJ))) AND (((temporal muscle) OR temporal fascia) OR ((((temporalis muscle) OR temporalis myofascial flap) OR Temporalis fascia) OR Temporalis superficial fascia)))) OR ((((interpositional arthroplasty) OR interposition arthroplasty)) AND ((temporomandibular joint) OR TMJ)))) AND ((((ankylosis) AND ((temporomandibular joint) OR TMJ))) AND ((((gap arthroplasty) OR condylectomy) OR osteoarthrectomy) OR ankylosis resection)) No limitation on language and publication date

82 items were found
